# Effects of psychosocial interventions on wellbeing in individuals with severe mental illness: a systematic review

**DOI:** 10.3389/fpsyg.2025.1574303

**Published:** 2025-03-26

**Authors:** David Johansson, Mikael Skillmark, Monika Allgurin

**Affiliations:** Department for Social Work, School of Health and Welfare, Jönköping University, Jönköping, Sweden

**Keywords:** eudaimonic, hedonistic, wellbeing, psychosocial interventions, severe mental illness (SMI), out-patient, systematic review

## Abstract

**Introduction:**

Wellbeing, encompassing hedonic and/or eudaimonic components, provides a two-dimensional framework for evaluating the effects of psychosocial interventions for individuals with severe mental illness (SMI). This study investigates how this conceptualization of wellbeing is reflected in existing research on psychosocial interventions for people with SMI. This is the first systematic review to assess the effects of psychosocial interventions on wellbeing as a purely positive phenomenon in this population. The study was registered in PROSPERO (CRD42024598954).

**Method:**

A systematic review was conducted on intervention studies involving adults with SMI receiving psychosocial interventions in an out-patient setting, with a control condition and a wellbeing outcome aligned with a wellbeing framework. Five databases were searched, supplemented by manual searches, yielding 2,842 potential studies. Due to considerable heterogeneity (*I*^2^ = 94%), interventions were analyzed independently, with results summarized based on the proportion of studies reporting significant effects. The study followed PRISMA guidelines.

**Results:**

Seventeen studies met the inclusion criteria. Only one study (6%) provided a full rationale for using a wellbeing measure as the primary outcome. Over 70% reported a significant positive effect on wellbeing. In 13 studies effect size could be calculated, 29% in reference to all 17 studies demonstrated a positive effect (ranging from small to large). Clinical implications of the wellbeing construct were discussed in 47% of the studies, including an increased emphasis on positive functioning. Fewer than 50% received a high-quality rating, and only three studies reporting significant effects used Intention-To-Treat (ITT) data.

**Conclusion:**

Research on two-dimensional wellbeing is a promising yet underprioritized field, providing a renewed focus on abilities and generating significant clinical implications. Wellbeing ought to be a prioritized outcome in out-patient treatment policies, but today no recommendation as to which interventions are most effective are possible due to insufficient data. The implications of detecting changes in wellbeing in individuals with SMI, along with recommendations for future research, are discussed.

## 1 Introduction

Nearly 20 years ago, the WHO (2005) called for a shift in how mental illness is addressed, highlighting that the predominant focus on symptom reduction had proven insufficient. More recently, Bickenbach et al. (2023) advocated for a functional revolution, urging researchers and clinicians to prioritize activities that people can engage in rather than focusing solely on treating specific deficits. For instance, a person on the autism spectrum will always have reduced mentalizing capacities (Baron-Cohen, 2008), yet they can still achieve a lived experience of health through meaningful activities (Bickenbach et al., 2023). Mental health is thus distinct from the mere absence of symptoms, meaning that positive functioning can coexist with symptoms of mental illness, which leads to a two-dimensional view of mental health (Westerhof and Keyes, 2010). Such a renewed concept of mental health may significantly benefit people with severe mental illness (SMI), sometimes also referred to as serious mental illness (Nevard et al., 2024), though the first term is more commonly used (Gonzales et al., 2022) and preferred here. Individuals with SMI experience persistent and severe psychological disabilities. In research, SMI most commonly refers to Schizophrenia and/or schizoaffective disorder when using a narrow definition. However, a broader functional definition, applied in this study, includes also other diagnoses (Gonzales et al., 2022). A functional definition of SMI typically includes a significant disability due to mental illness with a duration of at least 2 years (Parabiaghi et al., 2006; Ruggeri et al., 2000). In addition to psychological disabilities, individuals with SMI often experience physical health issues (Mitchell et al., 2013) and reduced life expectancy (Chesney et al., 2014). Due to their vulnerability, individuals with SMI are one of the prioritized groups within The United Nation’s Sustainable Development Goal 3 (SDG3) “Ensure healthy lives and promote well-being for all at all ages” (United Nations, 2015).

Wellbeing, as a definition of mental health, is currently an ambiguous concept in the scientific literature (Slade and Schrank, 2017), referring both to various models of positive functioning (Oades and Mossman, 2017) and the absence of symptoms of illness (Hanssen et al., 2023). The renewed conceptualization of mental health brings forward the concept of wellbeing as a form of subjective mental health that follows the “build-what’s strong” approach (Duckworth et al., 2005, p. 631). This perspective, rooted in positive psychology (Seligman, 2011; Slade, 2010), draws on two philosophical traditions dating back to ancient Greece: hedonistic wellbeing, which focuses on emotional wellbeing, and eudaimonic wellbeing, which emphasizes positive functioning (Ryan and Deci, 2001). However, currently, much research assesses mental health–and even subjective mental health–using mixed constructs and capacities (Fernández-Abascal et al., 2021; Zhang et al., 2024). For example, it’s common to conflate wellbeing with what Bickenbach et al. (2023) term capacities–psychological attributes such as executive functions (Goldberg, 2017), memory (Kolb and Whishaw, 2009), self-efficacy (Bandura, 1997), and mindfulness (Kabat-Zinn, 2015). While these capacities may enhance wellbeing and share overlapping items, they should not be used as substitutes for measures of wellbeing. Such examples raise concerns about the epistemological foundations of mental health assessment, particularly in individuals with SMI, where meaningful changes in wellbeing may go undetected.

The two-dimensional concept of wellbeing for individuals with SMI requires therefore a precise measurement. Scales that combine both positive and negative health constructs, such as most Quality-of-Life measures (Seow et al., 2019) or one-dimensional Recovery scales (Shanks et al., 2013) along with the numerous mental illness measures, are unsuitable for this purpose. To accurately assess mental health in individuals with SMI and avoid issues, such as ceiling effects (Bech et al., 2003), attentional bias (Beck, 2008) toward illness, and mood-congruent bias (Brewin et al., 1993) introduced by questions about illness, scales focusing exclusively on positively worded wellbeing constructs are preferred. The examples of models focusing solely on positive aspects are: Seligman’s (2018) PERMA model (positive emotions, engagement, positive relationships, meaning and accomplishment), Ryff’s (1989) psychological wellbeing (autonomy, personal growth, positive relationships, environmental mastery, purpose in life and self-acceptance) and Keyes (2005) subjective wellbeing (psychological, emotional and social wellbeing).

The currently dominating understanding of mental health in individuals with SMI is the Recovery perspective. This perspective serves as a collective term for efforts aimed at helping individuals with SMI returning to a fulfilling and satisfying life (Anthony et al., 1993), advocating concepts aligned with wellbeing, such as connectedness, meaning and purpose (Leamy et al., 2011). However, the concept of recovery, even when framed as personal recovery, remains indisputably one-dimensional. Recovery is invariably understood in relation to illness (Slade and Wallace, 2017), which may lead to reduced expectations regarding what, for example, a purposeful life can entail. Both professionals working with individuals with SMI and the individuals themselves often carry stigmatized perceptions about their abilities (Perkins et al., 2018).

In this article, we adhere to the two-dimensional concept of wellbeing to investigate how psychosocial interventions delivered in an out-patient context can affect wellbeing in individuals with SMI. Psychosocial interventions encompass a broad range of approaches that address the psychological and/or social aspects of an individual’s life, rather than focusing primarily on biological factors (Smart et al., 2020). Psychosocial interventions can thus be anything from one-to-one therapy to group activities.

While the effects of positive psychological interventions (PPI) have been previously reviewed (Geerling et al., 2020), the conceptualizations of wellbeing in this review included measures with mixed constructs. Similarly, Igarashi et al. (2021) examined common concepts in psychosocial interventions for individuals with SMI, identifying five previous reviews that included the wellbeing concept and one that addressed psychological functioning. However, none of these studies used measures of wellbeing that adhere to a positively formulated hedonistic and/or eudaimonic wellbeing framework, as applied here. Thus, no existing reviews assess the impact of psychosocial interventions on wellbeing in individuals with SMI within the epistemological framework outlined in this study. Since wellbeing-focused interventions may serve as an important complementary aspect to traditional treatment goals focused on minimizing illness in individuals with SMI, this study is of high relevance to inform future policies on treatment outcomes. It reinforces the need to prioritize wellbeing in future policies by advising on how a wellbeing framework could have broader clinical implications for how treatment is delivered and perceived by the recipients. This review is guided by the following research questions:

*RQ1*: How is wellbeing addressed in the rationale and design of the included studies?

*RQ2*: What are the reported effects of psychosocial interventions in outpatient treatment on wellbeing of adults with SMI?

*RQ3*: What are the reported clinical implications of psychosocial interventions focused on enhancing wellbeing for adults with SMI?

## 2 Method

This systematic review adheres to the recommendations outlined in Cochrane’s Handbook for Systematic Reviews edited by Higgins et al. (2019) and is reported in accordance with the Preferred Reporting Items for Systematic Reviews and Meta-Analyses (PRISMA) (Liberati et al., 2009; Page et al., 2021). The study was registered in PROSPERO (CRD42024598954).

### 2.1 Eligibility criteria

Population: Participants aged 18 years or older with SMI were included. The classification of a condition as SMI was based on one or more of the following indicators: diagnoses previously defined as SMI in research (Gonzales et al., 2022), reported duration and severity of mental illness, and/or whether the condition is described in the *Diagnostic and Statistical Manual of Mental Disorders* (DSM-5-TR) as likely to cause significant disability over an extended duration (Parabiaghi et al., 2006). Individuals with intellectual disabilities, biological brain damage such as stroke, or neurocognitive disorders were excluded. Other exclusion criteria included restraining circumstances like physical disabilities, imprisonment, and/or fugitive/asylum status.

Intervention: Psychosocial interventions delivered in an out-patient context were included. Studies involving co-occurring changes in medication were excluded.

Comparison: Eligible studies were required to use a randomized controlled trial (RCT) design or a quasi-experimental (QE) design. Control conditions were classified as active if participants received any form of treatment beyond monitoring; otherwise, they were classified as passive.

Outcome: In all potential studies the measures were assessed within the wellbeing framework and considered eligible if they measured only wellbeing components using multiple items (>1) and contained exclusively positive formulations. The only exception to this positivity criterion is Ryff’s (1989) original measure, which however is established as one of the most utilized instruments of psychological wellbeing. At least one of the following measures of wellbeing was required for inclusion: Adult State Hope Scale (HS) (Snyder et al., 1991); Flourishing Scale (FS) (Diener et al., 2010); Psychological Well-Being Scales (PWBS) (Ryff, 1989); Satisfaction with Life Scale (SWLS) (Diener et al., 1985); Warwick-Edinburgh Mental Wellbeing Scale (WEMWBS) (Tennant et al., 2007); WHO-5 Well-Being Index (WHO-5) (Topp et al., 2015).

Study characteristics: Included studies were required to be written in English, peer-reviewed and published in a non-predatory journal.

### 2.2 Information sources and search strategy

Five databases were searched on February 23, 2024, using iterative searches, successively adding each part of the PICO structure (see the protocol for the full search string). MEDLINE (EbscoHost) was searched using advanced search with Boolean phrases. PsycINFO (ProQuest) was searched using advanced search with Tiab and IF. CINAHL (Ebscohost) was searched using advanced search with Boolean/Phrase. Scopus (Elsevier) was searched within title, abstract and keywords. Web of Science (Clarivate) was searched using advanced exact search. Additionally, the reference lists of the included studies from the initial search were manually reviewed in July 2024 to find additional relevant studies.

### 2.3 Selection process

All retrieved titles and abstracts were independently screened by two researchers, and studies with the potential to meet the eligibility criteria proceeded to full-text assessment. The complete eligibility criteria based on the study’s PICO were independently used by two researchers to assess all full-text articles. Disagreements occurring at any stage of the selection process were resolved through discussion among all three authors. This process was repeated for the additional studies identified in the manual reference list search. All study screening and eligibility assessment were conducted using Covidence software.

### 2.4 Data collection process and data items

Data was extracted by two independent researchers following the published extraction protocol, with any disagreements resolved through discussion among all three researchers. The protocol was finalized after a pilot assessment, where all three authors independently extracted data from the same study.

Extracted data: country and author; design; type of groups; aim and RQs; primary outcome; measures of wellbeing; inclusion and exclusion criteria; total sample size; attrition; reason for withdrawals; overall mean age (SD); diagnosis; severeness rating; duration of illness (majority); type of intervention and control; total duration; main content of intervention and frequency; statistics from measures of wellbeing at baseline (T1), end of treatment (T2) and follow-up (T3); use of wellbeing justified theoretically by referring to theoretical references and/or empirically by referring to studies that have shown effects of wellbeing in the extracted articles introduction section (extracted as yes/no and if provided, describe); clinical implications of using wellbeing discussed in the extracted articles (extracted as yes/no and if provided, describe). All extracted data were recorded in Covidence software.

### 2.5 Quality assessment (risk of bias)

Quality assessment (QA) was made independently by two researchers in Covidence, with any disagreements resolved through discussion among all three researchers. The assessment utilized a tool with 17 criteria, as described in Olsson and Sundell (2023). This tool is endorsed by the CONSORT Statement (Moher et al., 2010), the TREND statement (Des Jarlais et al., 2004) and guidelines from Prevention science (Flay et al., 2005). Decisions for each criterion were categorized as “High” if present, “Low” if absent or “Irrelevant” (see all 17 items in the study’s protocol).

Each study could receive a score based on the number of “High” ratings, ranging from 0 to 16, as one QA category is always deemed irrelevant depending on the study design. Studies were classified based on scoring thresholds influenced by Olsson and Sundell (2023): high quality (≥12), medium quality (6–11), or low quality (<6). All QA decisions were made at the study level.

### 2.6 Synthesis methods

Data extracted for RQ1 was summarized in a table organized by the number of items each study provided for the rationale of using the concept of wellbeing. For studies with the same number of items checked, alphabetical order was applied. The results were then reported with the percentage of all studies. For RQ2, the interventions effects on wellbeing were analyzed using both a Forest plot, which displayed a standardized comparison of the individual interventions effects using Hedges’ *g* (McKenzie et al., 2019) among studies that provided sufficient descriptive data, and a narrative summary of all results, including both the calculated and reported findings. Percentages were used to report the relative frequency of findings across all 17 studies. Descriptive denominators such as type of control condition, intervention length, ITT data and QA were used in the narrative summary. An investigation of the heterogeneity among the interventions proved it to be considerable (*I*^2^ = 94%), excluding the possibility of a sound meta-analysis.

The Forest plot was generated from the results of a general linear mixed-effects model (GLMM) that included effect sizes, confidence intervals, and weights based on inverse variance using the Metafor package (Viechtbauer, 2010) in R (Version: 4.4.2). To prepare data for the GLMM, a global mean and standard deviation (SD) were calculated for both the experimental and control groups by averaging sub-indexes of wellbeing and/or the results from two wellbeing measures (McKenzie et al., 2019), these calculations were performed using SPSS (Version 29). In studies with more than one experimental condition, the condition that yielded the highest intervention effect was utilized (Table 1 identifies the conditions used for comparisons). The results from the last measure point were used, and the Forest plot was organized based on the type of control condition.

**Table 1:** Study characteristics.

Study	*N* (% females)	Diagnose	Type of intervention	Group or individual	Duration (months): T1–T2, T1–T3 (intensity)	Design	Control (Active vs. Passive)	Wellbeing outcomes	Data	QA
Carl et al. (2020)	256 (68)	Generalized anxiety disorder.	Cognitive Behavioral Therapy (CBT).	I	1.5, 2.5 (promotes daily use)	RCT	WL (P)	WEMWBS	M, SD (ITT)	High
Chaves et al. (2017)	96 (100)	Depression/dysthymic disorder.	Positive Psychology Intervention (PPI).	G	2.5, N/A (weekly, 2 h)	QE	CBT (A)	PWBS, SWLS	M, SD (ITT)	Medium
Cuijpers et al. (2022)^1^	680 (70)	Depression.	Step-by-Step: Psycho-education, behavioral activation, and PPI mechanisms.	I	1.25, 4.25 (weekly)	RCT	ETAU (P)	WHO-5	M, SD	High
Davidson et al. (2004)^2^	260 (57)	Psychotic disorder (50%), Affective disorder (34%), Anxiety disorder (2%), other axis I disorder (1%), unknown (12%), co-occurring substance use disorder (44%).	Social support to engage in social and/or recreational activities with peer and financial support.	I	4, 9 (weekly, 2-4 h)	RCT	Only financial support (P)	PWBS	M, SD (ITT)	Medium
Farquharson and MacLeod (2014)	82 (54)	Schizophrenia (43%), Bipolar (27%), Mood disorder (23%) and other (7%).	Goal setting and planning skills (GAP).	G	1, 2 (weekly, 2 h)	RCT	WL (P)	SWLS	M, SD	Low
Freeman et al. (2014)	30 (33)	Schizophrenia (74%), Schizo-affective disorder (20%), Delusional disorder (3%), Psychosis NOS (3%).	CBT + TAU.	I	2, 3 (<weekly, 1 h)	RCT	TAU (P)	WEMWBS	M, SD (ITT)	High
Halverson et al. (2021)	38 (47)	Early stages of Schizophrenia spectrum disorder.	Integrated Coping Awareness Therapy (I-CAT) + TAU.	I	9, 12 (14–24 weekly sessions)	RCT	TAU (A)	PWBS	LSM, SE	High
Harmanci and Budak (2022)	160 (46)	Schizophrenia.	Psychoeducation + TAU.	G	1.5, N/A (2 weekly)	CRCT	TAU (P)	FS	M, SD	Medium
Jensen et al. (2019)	198 (45)	Schizophrenia (76%), Bipolar disorder (24%).	Illness management and recovery (IMR) + TAU.	G	9, 21 (weekly)	RCT	TAU (A)	HS	M, SD (ITT)	High
Kızılırmak Tatu and Demir (2021)	45 (38)	Schizophrenia.	Psychoeducation	G	2, 5 (weekly, ≈1 h)	QE	TAU (P)	FS	Mdn, min-max	Medium
Lovell et al. (2018)	604 (59)	Depression (47%), Anxiety (32%), Bipolar (25%), Schizophrenia (23%), Personality disorder (17%), Panic disorders (9%), Eating disorder (6%) and Phobia (5%).	Conversational aid to enhance shared decision making in care planning +TAU.	I	6, N/A (≈12 contacts)	CRCT	TAU (P)	WEMWBS	M, SD (ITT)	High
Priebe et al. (2015)^1^	179 (31)	Schizophrenia (79%), Delusional disorders (1%), Schizoaffective disorders (13%), Unspecified non-organic psychosis (2%), Bipolar disorder (2%), Major depressive episode (2%).	DIALOG+ (patient rating of life domains guides session with care coordinators).	I	6, 12 (≈1/month)	CRCT	Perform the ratings, but at the end of session with the co-ordinator (P)	WEMWBS	M, SD	High
Sylvia et al. (2013)	66 (52)	SMI.	Exercise program.	G	<1, N/A (mean attending 3, 50 min groups)	QE	Psychoeducation (A)	PWBS	Mean change	Low
Tomba et al. (2017)	250 (100)	Anorexia nervosa (31%), Binge-eating disorder (34%), Bulimia nervosa (17%), Eating disorder not otherwise specified (18%).	CBT and nutritional rehabilitation program.	I	≈12, N/A (weekly 1 h CBT + 1 h with nutritionist)	QE	healthy controls	PWBS	M, SD (ITT)	Medium
Valiente et al. (2022)	141 (41)	Schizophrenia (72%), Affective disorders (9%), Anxiety disorders (6%), Personality disorders (8%), Others (5%).	PPI + TAU.	G	2.75, N/A (weekly, 1.5 h)	RCT	WL + TAU (A)	PWBS, SWLS	M, SD	High
Williams et al. (2019)	59 (51)	Schizophrenia (20%), Substance abuse disorder (25%), Bipolar disorder (19%), Depression (25%), Post traumatic stress disorder (14%), Other anxiety disorders (27%), Personality disorder (5%), Autism spectrum disorder (7%), Comorbid diagnoses 40%.	Choir.	G	12, N/A (10 week terms with 2 break weeks, each session 2.5 h)	QE	Creative writing (A)	WEMWBS	MLM	Low
Özdemir and Kavak Budak (2022)^2^	156 (23)	Schizophrenia.	Mindfulness-Based Stress reduction (MBSR).	G	2, 4 (weekly, ≈ 30 min)	CRCT	TAU (A)	FS	M, SD	Medium

^1 = have done ITT effect analyses, but only provide descriptive none ITT data; 2 = also have a second experimental condition not used here; CRCT, cluster randomized trial; ETAU, enhanced treatment as usual; FS, The Flourishing Scale; HS, Adult State Hope Scale; ITT, Intention-to-treat analysis; LSM, least squares means; M, mean; Mdn, median; MLM, multilevel modeling; NOS, not otherwise specified; PWBS, Psychological Well-Being Scales; SD, standard deviation; SE, standard error; SMI, e.g., major depression, bipolar disorder, schizophrenia; SWLS, Satisfaction with Life Scale; T1, baseline; T2, end of treatment; T3, follow-up; QA, Quality assessment; QE, Quasi-experimental trial; RCT, randomized controlled trial; WEMWBS, Warwick-Edinburgh Mental Wellbeing Scale; WHO-5, WHO-5 Well-Being Index; WL, wait list.^

RQ3 was analyzed narratively by summarizing the content of clinical implications of using wellbeing as discussed in the reviewed studies, with the most frequently reported implications presented first.

## 3 Results

### 3.1 Study selection

**Figure 1:**
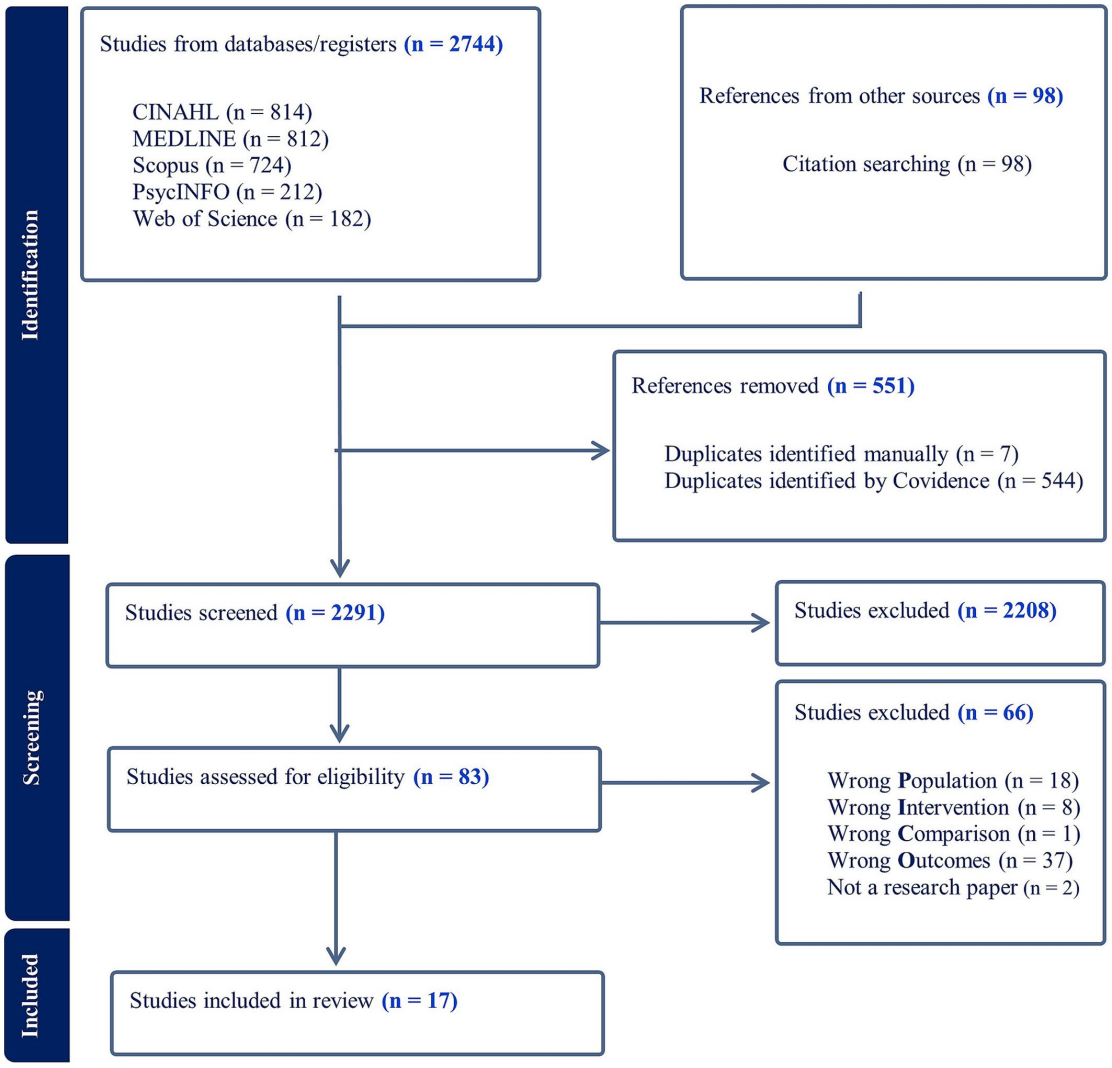
PRISMA flow diagram of study selection.

A total of 2,842 studies were identified for potential inclusion in the systematic review. After removing duplicates, 2,291 titles and abstracts were screened, and 83 studies were deemed eligible for full-text screening. Ultimately, 17 studies were included in the final analysis. The study selection process is summarized in Figure 1.

### 3.2 Study characteristics

The general characteristics of the 17 included studies are summarized in Table 1. Population sizes varied, with sample sizes ranging from 30 to 680, age ranges from 18 to 65+, and the proportion of females ranging from 23 to 100%. The duration of mental illness and/or mental health service contact was reported in eight studies (47%), with the mode exceeding 3 years.

Interventions were multifaceted, with Cognitive behavioral therapy (CBT) and Psychoeducation serving as the core feature in three studies each (18%). Positive psychology interventions (PPI) were the core feature in two studies (12%), while the remaining interventions incorporated unique or mixed core elements. A brief description of each intervention is provided in Supplementary Table S1. Eight studies (47%) utilized individual sessions, while nine (53%) offered group sessions. Face-to-face sessions were conducted in the majority of the interventions (15 studies 88%), while the remaining two were delivered digitally (Carl et al., 2020; Cuijpers et al., 2022). The duration of the interventions was short (≤3 months) in 10 studies (59%), medium (4 to 6 months) in three studies (18%) and long (≤9 months) in four studies (23%). Follow-up measurements were provided in nine studies (53%).

Five studies (29%) had an attrition rate between 0 and 10% (Carl et al., 2020; Freeman et al., 2014; Harmanci and Budak, 2022; Kızılırmak Tatu and Demir, 2021; Priebe et al., 2015), six studies (35%) between 11 and 25% (Chaves et al., 2017; Halverson et al., 2021; Lovell et al., 2018; Sylvia et al., 2013; Tomba et al., 2017; Özdemir and Kavak Budak, 2022), two studies (12%) between 26 and 50% (Farquharson and MacLeod, 2014; Jensen et al., 2019), and three studies (18%) that had >50% attrition (Cuijpers et al., 2022; Valiente et al., 2022; Williams et al., 2019). Davidson et al. (2004) did not report the attrition.

Comparisons were conducted using three different designs: eight studies (47%) employed a randomized controlled trial (RCT) design, five utilized quasi-experimental designs (QE) (29%) and four used cluster randomized controlled trial (CRCT) design (24%). The control conditions consisted of active treatments in seven studies (41%) and passive/monitoring conditions in nine studies (53%), while one study (6%) compared participants to healthy controls.

Outcomes related to wellbeing were measured using the Psychological Well-Being Scale (PWBS) in six studies (35%), the Warwick–Edinburgh Mental Well-being Scale (WEMWBS) in five studies (29%), and The Flourishing Scale (FS) in three studies (18%). The Satisfaction with Life Scale (SWLS), Adult State Hope Scale (HS) and WHO-5 Well-Being Index (WHO-5) were each used in one study (6%). In two studies (12%), SWLS was used in combination with PWBS. Descriptive data were presented as mean (M) and standard deviation (SD) in 13 studies (76%), and intention-to-treat (ITT) analysis was employed in seven studies (41%).

Considering the quality assessment (QA), eight studies (47%) were rated as high quality, six studies (35%) as medium quality, and three studies (18%) as low quality.

### 3.3 How is wellbeing addressed in the study rationale and design?

One study (6%) addressed all criteria for the rationale of using wellbeing, including providing a theoretical justification, empirical justification, inclusion in the aim/RQ and using wellbeing as a primary outcome measure (see Table 2). Seven studies (41%) provided a theoretical justification, empirical justification and included wellbeing in the aim/RQs. Freeman et al. (2014) was the only study (6%) to include both a theoretical and empirical justification for wellbeing without incorporating it into the aims/RGs. Similarly, Kızılırmak Tatu and Demir (2021) included a theoretical justification and incorporated wellbeing in the aim/RQ but lacked empirical justifications (6%). Three studies (18%) addressed wellbeing in their aims/RQs without additional justifications. Finally, four studies (24%) included a measure of wellbeing but did not provide any justifications for its inclusion.

**Table 2:** Rationale for including the wellbeing concept.

Study	Theoretical justification of the use of wellbeing (yes/no)	Empirical justification of the use of wellbeing (Yes/No)	Wellbeing in RQ and/or aim (Yes/No)	Primary outcome
Valiente	Yes	Yes	Yes	PWBS, SWLS
Chaves	Yes	Yes	Yes	BDI-II
Farquharson	Yes	Yes	Yes	–
Halverson	Yes	Yes	Yes	mDES, QLS, FESFS, PSS
Harmanci	Yes	Yes	Yes	–
Tomba	Yes	Yes	Yes	–
Williams	Yes	Yes	Yes	–
Özdemir	Yes	Yes	Yes	–
Freeman	Yes	Yes	No	BCSS, GPTS
Kızılırmak Tatu	Yes	No	Yes	–
Carl	No	No	Yes	GAD-7
Davidson	No	No	Yes	–
Sylvia	No	No	Yes	–
Cuijpers	No	No	No	PHQ-9, WHODAS-12
Jensen	No	No	No	GAF-F
Lovell	No	No	No	HCCQ-10
Priebe	No	No	No	MANSA

BCSS, Brief Core Schema Scales; BDI-II, The Beck Depression Inventory-II; FESFS, The First Episode Social Functioning Scale; GAD-7, Generalized anxiety disorder 7-item; GAF-F, Global Assessment of Functioning; GPTS, Paranoid Thoughts Scale; HCCQ-10, the Health Care Climate Questionnaire 10-items; MANSA, Manchester Short Assessment of Quality of Life; mDES, The modified self-report Differential Emotion Scale; PHQ-9, The Patient Health Questionnaire 9-item; PSS, The Perceived Stress Scale; QLS, The abbreviated Quality of Life Scale; WHODAS-12, WHO Disability Assessment schedule 12-items.

All 10 studies with a theoretical rationale for wellbeing (59%) underscored how psychological functioning can have a positive impact on individuals. For example, Halverson et al. (2021) referenced Fredrickson’s (2001) “broaden-and-build” theory of positive emotions, which expands the behavioral repertoire. Four of them (24%) emphasized two-dimensionality of wellbeing, highlighting that it is distinct from merely the absence of symptoms (Chaves et al., 2017; Tomba et al., 2017; Valiente et al., 2022; Williams et al., 2019).

Empirical justifications were provided by nine studies (53%), demonstrating how enhanced wellbeing positively influenced other outcomes, such as improving quality of life (Harmanci and Budak, 2022) and alleviating depression (Chaves et al., 2017).

### 3.4 What are the reported effects of psychosocial interventions in outpatient treatment on the wellbeing of adults with severe mental illness (SMI)?

**Figure 2:**
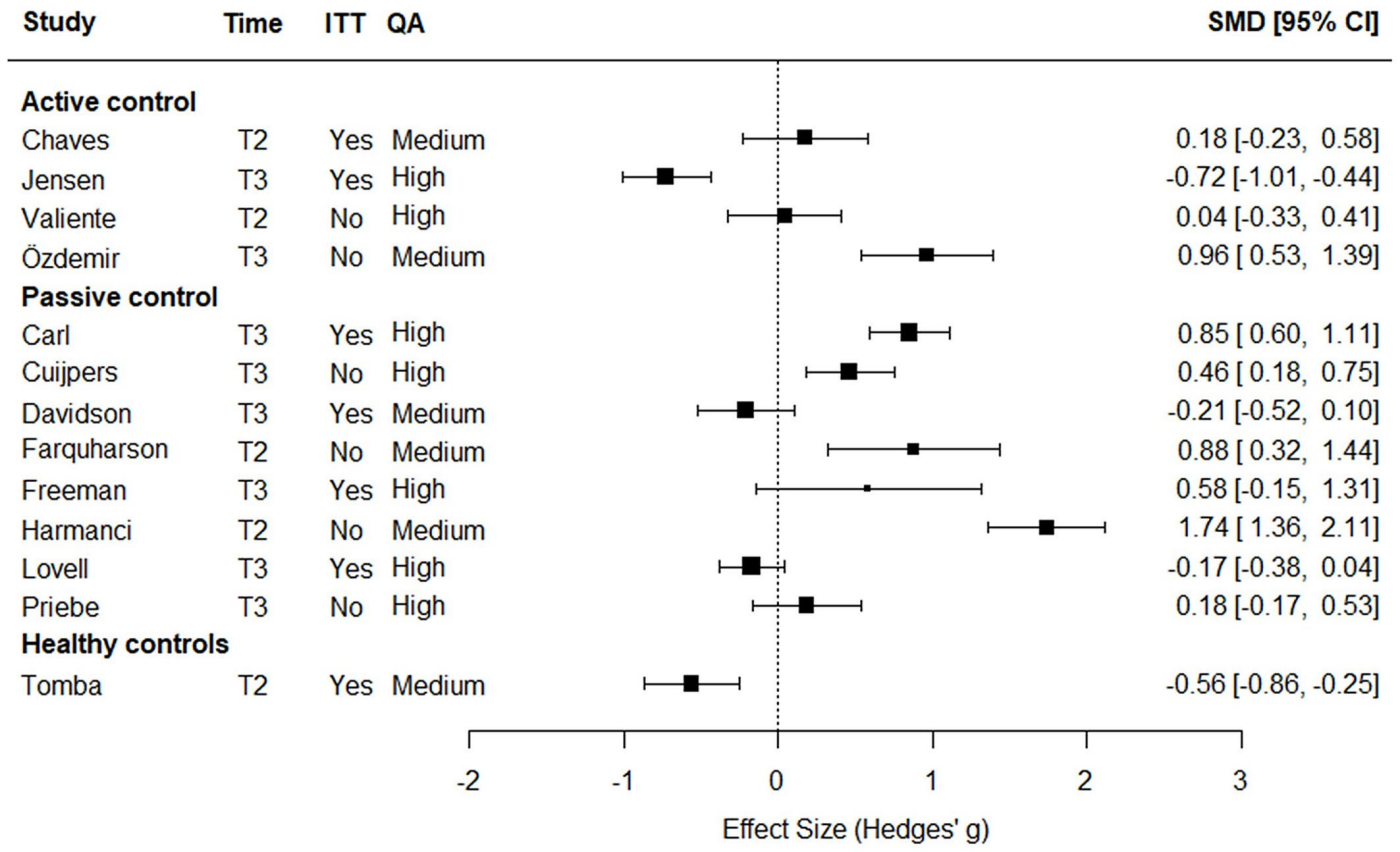
Weighted individual intervention effects on wellbeing by type of control condition.

The 13 studies (76%) that reported descriptive outcome data at T2 or T3 are included in Figure 2. Five studies (29%) demonstrated a positive effect on wellbeing with a 95% confidence interval (CI) entirely on the positive side, three of them were group interventions. The effect sizes for these studies ranged from 0.46 to 1.74, representing small to large effects (Cohen, 1988). Two studies, Carl et al. (2020) and Cuijpers et al. (2022), received high QA and weighting, showing large and small effect sizes, respectively, at T3. All five studies utilized short-duration interventions (<3 months). The two studies with the largest effect sizes – 0.96 and 1.74 (Harmanci and Budak, 2022; Özdemir and Kavak Budak, 2022) – were of medium quality, group interventions, and did not provide descriptive ITT data. Among these five studies with positive effects, four employed passive control conditions.

None of the interventions lasting 4 months or longer showed a reliable positive effect on wellbeing in the calculated effect sizes. Both Jensen et al. (2019), which utilized an active control, had high QA, and weighting, and Tomba et al. (2017), which used healthy controls and had a medium QA, demonstrated negative effects at T3 and T2, respectively. Both interventions, employing ITT data, had long durations of 9 and 12 months, respectively.

Four studies (24%) did not provide sufficient descriptive data for the effect size calculations in Figure 2 (Halverson et al., 2021; Kızılırmak Tatu and Demir, 2021; Sylvia et al., 2013; Williams et al., 2019). Among these, Kızılırmak Tatu and Demir (2021), a medium-quality study, with a group intervention reported a significant treatment effect in the experimental condition after a short-duration intervention compared to a passive control. However, ITT data were not used to measure wellbeing at T3. Three studies (18%) utilized active contol conditions where the group-by-time interaction did not show a significant effect on wellbeing in the experimental condition. However, Chaves et al. (2017), Williams et al. (2019), and Tomba et al. (2017) all reported a significant effect of time on wellbeing. In Chaves et al. (2017) and Williams et al. (2019) both the experimental and control conditions showed significant increases in wellbeing. Similarly, participants in the intervention condition in Tomba et al. (2017) demonstrated a significant positive change in wellbeing from T1.

In summary, five studies in Figure 2 showed a positive effect on wellbeing (29%). Among these, three were group interventions (18%), two had high QA (12%), one had an active control (6%), and one used ITT data (6%). One study in Figure 2, which employed an individual intervention design, demonstrated a positive effect on wellbeing (6%), had high QA and used ITT data. None of the group interventions achieved this combination. A total of 12 studies reported a significant effect on wellbeing (71%), of which seven were group interventions (41%), five had high QA (29%), five had active control conditions (29%), and three used ITT data (18%). Two studies employing an individual intervention design reported a significant effect on wellbeing (12%), both with high QA and ITT data–again, a combination not achieved by any group interventions.

### 3.5 What are the reported clinical implications of interventions focused on enhancing wellbeing for adults with SMI?

Clinical implications of using wellbeing constructs were discussed in eight studies (47%) (Chaves et al., 2017; Farquharson and MacLeod, 2014; Freeman et al., 2014; Kızılırmak Tatu and Demir, 2021; Tomba et al., 2017; Valiente et al., 2022; Williams et al., 2019; Özdemir and Kavak Budak, 2022). In five studies (29%), it is reported that framing interventions from a wellbeing perspective could lead clinicians to focus more on positive aspects of functioning rather than solely minimizing symptoms (Farquharson and MacLeod, 2014; Freeman et al., 2014; Valiente et al., 2022; Williams et al., 2019; Özdemir and Kavak Budak, 2022).

The potential for destigmatizing treatment was discussed in two studies (12%) (Chaves et al., 2017; Williams et al., 2019). Additionally, two studies (12%) highlighted the impact of targeting wellbeing on adherence to treatment (Kızılırmak Tatu and Demir, 2021; Tomba et al., 2017).

## 4 Discussion

The functional revolution, as advocated by Bickenbach et al. (2023), implies a shift toward wellbeing, prioritizing measures, theories and empirical findings that support wellbeing in individuals with SMI. The results of our study indicate that we are yet to witness such a shift to come.

This first research question (RQ) examined how wellbeing was addressed in the rationale of the studies. However, only one study–representing 6% of the reviewed studies–met all four criteria for a rationale supporting the investigation of wellbeing. Notably, nearly 25% of the studies provided no rational for including a wellbeing measure. Measuring a phenomenon without a clear purpose risks inviting *post hoc* interpretations of unexpected effects. While this could spur intriguing new research questions, it may also result in sample-specific findings that are difficult to replicate (Open Science Collaboration, 2015).

With one exception, the reviewed studies predominantly employed a primary outcome that framed mental health as the reduction of illness symptoms–a perspective that is incompatible with a wellbeing-oriented approach that rethinks what mental health is. Wellbeing is conceptually referred to in 59% of the reviewed studies. However, only one study uses a primary outcome that measures wellbeing in alignment with the positively oriented measure of wellbeing. Individuals with SMI often experience persistent psychological disabilities (Parabiaghi et al., 2006; Ruggeri et al., 2000), making it essential to use outcomes that are not constrained by co-existing disabilities to capture changes in their level of functioning.

Furthermore, it is only the primary outcome of the study that can be adequately powered. Secondary outcomes are inherently at greater risk of both Type I and Type II errors, making their results preliminary at best (Andrade, 2020). Ensuring adequate power is crucial, particularly in studies involving individuals with SMI, as these studies often encounter high attrition rates and require substantial efforts to recruit participants (Kanuch et al., 2016). In this review, 71% of the studies reported 10% attrition rate or higher, with 18% experiencing attrition rates exceeding 50%. Given that attrition rates above 10% demand special care to mitigate the risk of bias (Twisk et al., 2020), prioritizing the selection of primary outcome becomes a critical consideration in research on individuals with SMI.

The second RQ examines the effects of interventions on wellbeing in individuals with SMI, showing that wellbeing ought to be given more priority in research. It is evident that the level of wellbeing can be improved in individuals with SMI. In the effect calculations performed here, 29% of the studies demonstrated a positive effect. Particularly, Harmanci and Budak (2022) reported a large effect on wellbeing through psychoeducation. Across all included studies, 71% reported significant positive changes in wellbeing. Based on the calculated effect sizes, the only intervention that should be cautioned against is Illness management and recovery (IMR) (Jensen et al., 2019), as it has a clear negative impact on wellbeing and due to high quality low risk of this result being an effect of bias. The authors also reference other studies on this intervention that show no positive effects, though these studies do not focus strictly on wellbeing. Additionally, IMR is a long intervention (9 months), requiring considerable time investment from both clients and professionals. Tomba et al. (2017) also show a negative effect in the calculated effect sizes, but this is largely explained by the comparison being made with healthy controls. It is unsurprising that a sample with SMI does not reach the same level of wellbeing as healthy controls.

Due to the substantial heterogeneity of the interventions, direct comparisons of effectiveness are avoided, as factors beyond the core features of the interventions may influence outcomes. Theoretically, certain interventions like PPI that formally intend to target wellbeing would be more effective in raising wellbeing, but this is not conclusively proven here. PPI interventions are not the most effective based on the calculated effect sizes. Both Chaves et al. (2017) and Valiente et al. (2022) utilized PPI interventions with active control conditions, but in the effect calculations, they did not demonstrate a reliable positive effect.

Hence, the type of control condition appears to influence outcomes. Among the calculated effect sizes, four of five studies showing a positive effect utilized a passive control condition, as did seven of the 12 studies reporting significant results. While using a passive control condition does not violate performance bias (unduly provision of treatment) (Jüni et al., 2001), the impact of active vs. passive control condition must be considered when interpreting results. However, this is not always accounted for (Karlsson and Bergmark, 2015). Offering active treatment in both the intervention and control conditions can generate expectations of positive effects (Geers and Miller, 2014) that differ from those elicited by passive control conditions. Although the interventions in this review are too heterogeneous to allow further analysis of control condition effects, studies with passive control conditions appear more likely to yield positive results, potentially inducing bias in favor of certain interventions. This reinforces the decision to avoid direct comparisons between interventions.

On the topic of bias, compared to all included studies, the studies showing a positive calculated effect here, 12% were assessed as high quality, and 6% included ITT. Furthermore, across all the studies, those that reported significant effects, 29% were assessed as high quality and 18% included ITT data. The first point to note is the low percentage of studies using ITT data. This highlights the challenge of addressing the effectiveness of interventions in changing the degree of wellbeing. Effectiveness, assessed using ITT data, evaluates an intervention’s impact on all participants commencing the intervention, whereas efficacy focus only on participants who complete it (Flay et al., 2005). This distinction is important because efficacy may introduce bias, as it reflects the preference of completers who may favor the intervention.

Another important point is that nearly 60% of the studies with positive effect were rated below high quality. Since quality here reflects the risk of bias, this finding underscores the need to interpret the effectiveness of individual interventions cautiously, as unrecognized bias could play a role.

With these limitations in mind, the synthesized results here reveal some discrepancies with previous research on wellbeing in individuals with SMI. None of the interventions lasting 4 months or longer achieved a significant effect on wellbeing and both the calculated effects and reported results indicate that group interventions more frequently achieved positive outcomes compared to individual interventions, though the difference is small (3 vs. 2 and 7 vs. 5, respectively). Reviews by Geerling et al. (2020) and Sin and Lyubomirsky (2009) on PPI suggest that longer interventions are needed to impact wellbeing in individuals with SMI. Additionally, Sin and Lyubomirsky (2009) conclude that individual interventions tend to be more effective. The apparent contradictions may stem from the strict epistemological framework applied here where no items relating to illness were allowed. Wellbeing levels in individuals with SMI may may be detected more more rapidly when they are not constrained by co-existing symptoms of illness. The epistemological implications for wellbeing also introduce the third RQ, which addresses the further clinical implications of utilizing the wellbeing concept.

Wellbeing seen through the framework applied here have been regarded as an academic and theoretical tradition that has made a limited impact on clinical practice (Slade, 2010). However, in this review, nearly half of the articles (47%) discuss implications of using wellbeing, indicating that it is beginning to have a meaningful clinical impact in research.

The most frequent reported implication (29%) is that it shifts clinicians toward recognizing positive aspects and strengths in individuals with SMI. Bickenbach et al. (2023) highlight that while individuals may experience reduced capacities in specific functions, they can still find meaning in activities, leading to a lived experience of health. For individuals with SMI, clinicians focusing on strengths rather than deficiencies are more likely to promote this sense of lived health by highlighting what they can achieve.

However, there are other reported positive implications worth considering, especially in the long run. Interventions utilizing wellbeing have been reported to possibly reduce stigmatization associated with treatment and/or increase the adherence to treatment (12%, respectively). As stigmatization is a pertinent issue for individuals with SMI (Hansson et al., 2011; Perkins et al., 2018), a reduction of this could spur an important change. Interventions focusing on wellbeing and positive aspects of functioning may reduce stigma by encouraging individuals and professionals to recognize abilities instead of disabilities. Individuals with SMI have had higher dropout rates from treatment (Hamilton et al., 2011). Treatment adherence and reduced stigmatization are likely interconnected pieces of the same puzzle, which could significantly improve long-term outcomes for individuals with SMI.

### 4.1 Limitations

The first limitation in this study concerns the definition of the population, which directly affects the development of the search string and inclusion criteria. The functional definition of SMI that exist uses thresholds for disability and duration, such as Global Assessment of Functioning (GAF) (Aas, 2010) scores of ≤50 and a disability duration of 2 years (experienced or prognosed) (Parabiaghi et al., 2006; Ruggeri et al., 2000). However, population characteristics in studies frequently do not provide this information in sufficient detail. The wider approach used here to define SMI by diagnoses previously recognized as SMI (Gonzales et al., 2022) and referencing diagnostic criteria in the DSM-5-TR (APA, 2022) includes more diverse diagnoses such as major depression (MD) and generalized anxiety disorder (GAD). Compared to a narrower SMI definition that only includes Schizophrenia and/or schizoaffective disorder, the broader definition may impact results yielding larger or different effects. In this review, only short-duration interventions produced positive effects–however, three of these included participants with Schizophrenia (Farquharson and MacLeod, 2014; Harmanci and Budak, 2022; Özdemir and Kavak Budak, 2022), and the largest effect was observed in a sample with Schizophrenia (Harmanci and Budak, 2022). Thus, while the broader SMI concept encompasses a heterogeneous group of diagnoses that may influence the results, it is not immediately clear how this would introduce bias in our results. Nevertheless, the variability in SMI definitions across studies presents a limitation for comparison.

The second limitation in this study concerns the use of a grand mean of wellbeing. There are different components in these measures and measures that have a more hedonistic perspective, like satisfaction measures (see for example WHO-5) may be more state dependent, when measures of psychological wellbeing are more trait dependent, and thus more stable (Weijers and Jarden, 2017). However, phenomenologically it is plausible to say that measures of positive constructs all tax a concept of wellbeing if they belong to the hedonistic and eudaimonic aspects of wellbeing. It is also the case that when perceived multifactorial measures of wellbeing, such as Keyes Mental Health Continuum, short form (MHC-SF) are validated, a single factor best represents the result (Lamers et al., 2011; Santini et al., 2020). All measures used by included studies, except PWBS, are according to their manual possible to use as a summary measure of wellbeing. Here, two included studies use both a hedonistic measure (WHO-5 and SWLS) and an eudaimonic measure (PWBS) and there is no significant difference in the reported results between the measures (Chaves et al., 2017; Valiente et al., 2022). The study with the largest calculated effect size in Figure 2 is primarily a measure of eudaimonic wellbeing (FS). The PWBS measure (Ryff, 1989) has six components of psychological wellbeing. When investigated separately, there was no clear trend as to which components of the Ryff PWBS are changed by the interventions. It is thus not possible to see an obvious trend in the data saying that a grand mean of wellbeing masks important effects when the RQ concerns effects on the wellbeing concept used here. Together with methodological considerations to avoid violation of the unit-of-analysis (McKenzie et al., 2019), that is to use the same participants repeatedly in separate analyses, only one effect size calculation can be done and then using a grand mean it the best solution.

The third limitation involves the calculated effect sizes. The data comprises both T2 and T3 data. Methodologically, as with measures that include subindexes, it is necessary to use a single data point to calculate the effect size and the recommended approach is to always use the final data point (McKenzie et al., 2019). Doing this will introduce heterogeneity in the data by comparing end-of-treatment data with follow-up data. The main limitation in this comes down to not being able to analyze the lasting effect of the interventions on wellbeing as the measure contains both acute post interventions effects and follow-up effects.

The fourth limitation of this review concerns the types of interventions included and the ability to evaluate their effects on well-being, as addressed in RQ2. As the outcomes instead of the type of intervention were the decisive eligibility criteria, thus resulting in a wide variety of interventions and considerable heterogeneity it is not possible to calculate a general effect of the interventions on wellbeing or compare the effectiveness of the interventions. Doing a meta-analysis with a summary measure of the average effect would be misleading (Deeks et al., 2019). Different sub-analyses were explored but did not yield a feasible solution. With more similar interventions using appropriate outcomes, future studies could categorize intervention types and conduct sub-analyses to identify the most effective approaches. The aim in RQ2 was to demonstrate that the degree of wellbeing can be changed, highlighting the importance of epistemology, rather than to prove the effect of a specific type of intervention, which would have required a different emphasis in study selection and analyses.

## 5 Conclusion

Research on wellbeing as a two-dimensional phenomenon for individuals with SMI is a promising yet underprioritized field. It is free from a focus on persistent disabilities and appears to generate more significant clinical implications. Among the reported results, nearly three-quarters of the interventions show a positive impact on wellbeing, and in the more standardized comparison with calculated effect sizes, almost one-third demonstrate a reliable positive effect. This underscores the importance of incorporating wellbeing as an outcome in out-patient treatment, following the epistemological framework suggested here.

While it is not possible to determine which intervention is most effective, the findings indicate that various interventions and modes of delivery may have a positive impact on wellbeing. As reported in the results section, only one type of intervention not only lacked positive effects on wellbeing but also had potential detrimental outcomes.

Detecting changes in wellbeing may have significant implications as psychological effects are associated with the results obtained from a measure: a positive result can induce a more optimistic mood, while a negative result may have further repercussions though mood-congruent bias (Brewin et al., 1993), where an individual’s current mood influences their perception and may extend to other areas of life. Similarly, attentional bias (Beck, 1964) describes how a positive result could increase the likelihood of an individual focusing on other positive aspects of life. The key takeaway is that identifying positive changes in individuals who have struggled with mental illness for extended periods is far from trivial and carries meaningful consequences. If this epistemological framework also increases the likelihood of detecting changes in wellbeing within shorter timeframes, it would further support its utility.

This review also identifies shortcomings in the field that limit the possible inferences and highlight areas that need to be addressed diligently in future research. Based on this systematic review, the following recommendations are proposed:

1. Provide a clear rationale for the use of wellbeing in the study to clarify the concept and its potential to the field of mental health for individuals with SMI.
2. Use wellbeing as the primary outcome when designing studies to ensure the power calculation for sample size is appropriate.
3. Employ measures of wellbeing that exclusively assess positive constructs within the framework of wellbeing.
4. Include an active control condition.
5. Use ITT data and provide descriptive ITT data.
6. Assess the statistical relationship between measures of wellbeing and measures of illness to further validate the two-dimensionality of mental health for individuals with SMI.
7. Provide a severity assessment of the population using a general instrument, such as the WHO Disability Assessment Scale (WHODAS) (Üstün, 2010), and include a report on the duration of experienced or prognosed disability.

Point 1–5 have been discussed above, and adhering to these recommendations would enhance the ability to draw inferences about which interventions are most effective. The effectiveness of interventions should be the decisive criterion, as only this can guide practice in selecting the most suitable interventions.

Point 6 identifies a gap in the research, as the two-dimensional nature of the wellbeing concept suggests that wellbeing can be improved independently of changes in symptoms of illness. However, of the included studies only Kızılırmak Tatu and Demir (2021) have analyzed this aspect and it was not included as a prioritized research question in their study. While previous research has demonstrated two-dimensionality of wellbeing in common illness (Westerhof and Keyes, 2010), it is arguably even more critical to investigate this phenomenon in the context of SMI due to the nature of these disabilities.

Point 7 would enhance the precision of the SMI concept and further validate its usefulness as an identified group for whom wellbeing may serve as an important complementary approach to symptom reduction. Given the challenges in accurately diagnosing psychiatric conditions (Plana-Ripoll et al., 2019) and determining appropriate treatments, wellbeing could serve as a universal intervention for individuals with more severe disabilities.

Rigorous and methodologically sound wellbeing research has the potential to provide valuable insights for both researchers and clinicians. Such work aligns with the aims of SDG3, to “Ensure healthy lives and promote well-being for all at all ages” (United Nations, 2015), thereby contributing to what Bickenbach et al. (2023) refer to as the functional revolution.

## Data Availability

The raw data supporting the conclusions of this article will be made available by the authors, without undue reservation.
