# Supplementary material for: Effects of psychosocial interventions on wellbeing in individuals with severe mental illness: a systematic review
Source: Front Psychol. 2025 Mar 26;16:1574303. doi: 10.3389/fpsyg.2025.1574303 (PMC11979722; doi:10.3389/fpsyg.2025.1574303)
Supplement: Supplementary file 1 [file Data_Sheet_1.pdf]

Table S1. Description of the Interventions Used.

Study	Intervention and Control	Modules/structure
Carl et al. (2020)	I = digital CBT, Daylight<br>C= Waitlist	The four modules (~10–20 min in length) are based on principles of applied relaxation; stimulus control; cognitive restructuring; and imaginal exposure.
Chaves et al. (2017)	I = PPI	Topics: (1) Treatment goals and depression overview, (2) Positive emotions, (3) Savoring, (4) Gratitude, (5) Relationships and kindness, (6) Self-compassion, (7) Personal strengths, (8) Life purpose, (9) Resilience, (10) Relapse prevention.
	C = CBT	Topics: (1) Goals, norms, and depression psychoeducation, (2-3) Behavioral activation, (4-6) Cognitive restructuring, (7-9) Social skills, (10) Relapse prevention.
Cuijpers et al. (2022)	I = Step-by-Step	The hybrid app intervention provided psychoeducation and behavioral activation training through an illustrated narrative, plus techniques like stress management, gratitude, positive self-talk, social support, and relapse prevention. Trained 'e-helpers' offered weekly support via phone or messages (up to 15 minutes).
	C = ECAU	ECAU included a one-page online psychoeducation sheet and referral list to evidence-based care, with app- or web-based delivery on depression and anxiety using text from Step-by-Step's first session for consistency.
Davidson et al. (2004)	I1 = Social support, consumer	Paired with a matched volunteer mental health 'consumer' in recovery, participants engaged in community social or recreational activities, each receiving a $28 monthly stipend.
	C = Only the financial incentive	To facilitate activities, they received a $28 stipend each month.
Farquharson and MacLeod (2014)	I = GAP<br>C = Waitlist	Goal setting and planning skills.
Freeman et al. (2014)	I = CBT and TAU	The agenda focused on: (1) negative self-thoughts, (2) positive activities, and (3) positive self-thoughts.
	C = TAU	Delivered per national and local guidelines, typically including antipsychotic prescriptions, community mental health visits, and regular outpatient psychiatrist appointments.

Halverson et al. (2021)	I = I-CAT +TAU	The manual-based intervention included collaborative agenda setting, skill practice, and homework: Part I covered stress reactivity and mindfulness; Part II combined mindfulness with coping strategies; Part III focused on personalized routines and progress tracking.
	C = TAU	TAU involved supportive therapy and evidence-based treatments for first-episode psychosis.
Harmanci and Budak (2022)	I = Psycho-education + TAU	Psychoeducation sessions covered: (1) empathy and health perceptions, (2) stigma, goals, and well-being, (3) future barriers and compliance, (4) change initiation through homework, (5) future planning and successes, and (6) health perception, adherence, and support.
	C = TAU	Patients continued with routine pharmacological treatments and CMHC practices.
Jensen et al. (2019)	I = IMR + TAU	Patients receiving IMR were provided educational handouts on 11 module topics: (1) Recovery strategies, (2) Mental illness facts, (3) Stress-Vulnerability Model, (4) Social support, (5) Medication use, (6) Substance use, (7) Relapse prevention, (8) Stress and problem coping, (9) Symptom management, (10) Navigating mental health services, and (11) Healthy lifestyles.
	C = TAU	Individually adapted interdisciplinary treatment containing medication, individual case manager support, individual and group therapy, and unstandardized psychoeducation.
Kızılırmak Tatu and Demir (2021)	I = Psycho-education	Sessions: (1) Preparation, (2) Recognizing schizophrenia, (3) Treatment evaluation, (4) Stress and coping, (5) Communication skills, (6) Problem-solving, (7) Interpersonal relationships and social activities, (8) Program evaluation.
	C = TAU	Routine follow-up (arranging treatment by the doctor, answering the patient's and family's questions about treatment) continued in the polyclinic.
Lovell et al. (2018)	I = shared decision-making	A conversational aid to help staff involve patients in decisions and integrate shared decision-making into mental health care planning.
	C = TAU	Not further described.
Priebe et al. (2015)	I = DIALOG+	Each DIALOG+ session starts with patients rating satisfaction on 8 life domains from a symptoms perspective and 3 treatment aspects (1-7 scale), with an option to request help. Scores are reviewed with clinicians, who offer positive feedback on improvements. A joint decision follows on domains to explore further, using a 4-step approach: (1) understanding concerns, (2) envisioning best outcomes,

		(3) exploring resources, and (4) setting actions, which are reviewed next session.
	C = Only answers the questions	Patients conducted the same ratings as in DIALOG+ using the devices and software, but at the end of the meetings, independently, and without further discussion.
Sylvia et al. (2013)	I = Exercise program	The trainer created individualized exercise programs with goals aligned to American College of Sports Medicine guidelines (5 days/week, 30 min, moderate intensity).
	C = Psycho-education	A psychoeducation group on wellness covered diet, exercise, medication adherence, and behavioral scheduling, with discussions on exercise importance and barriers.
Tomba et al. (2017)	I = CBT and nutritional rehabilitation program	Cognitive-behavioral techniques (e.g., cognitive restructuring, assertiveness training, self-monitoring, behavioral homework, food exposure) combined with nutritional rehabilitation (e.g., psychoeducation on nutrition, weight restoration, health impacts). Daily case discussions between therapists and nutrition physicians tailor sessions to emerging needs.
	C = Healthy controls	No intervention
Valiente et al. (2022)	I = PPI + TAU	1. Welcome/Intro, 2. Positive emotions, 3. Regulating negative emotions, 4. Gratitude, 5. Anger & forgiveness, 6. Self-compassion, 7. Strengths, 8. Positive relationships, 9. Values & purpose, 10. Resilience, 11. Maintenance & farewell.
	C = TAU + waitlist	Psychiatric rehabilitation aims to maximize personal and social autonomy through individualized plans, including social skills training, coping strategies, daily living activities, and cognitive rehabilitation.
Williams et al. (2019)	I = Choir	The choir, led by an experienced conductor, meets regularly for rehearsals focused on learning songs by ear, with no musical experience required. Rehearsals emphasize warm-ups, social connection, and mutual support, likening the choir to a "family." After each session, members socialize over lunch. Performances around Brisbane fostered hope, empowerment, and purpose, with members wearing uniform shirts to promote unity.
	C = Creative writing	In class, participants sat in a square setup, received writing tips, completed exercises, and were given weekly homework. They bonded before class, shared their work aloud, and provided respectful feedback, with the option to write fiction or personal stories. The group fostered hope and empowerment, aiming to build

		skills for personal projects. At year-end, participants presented their work alongside choir performances.
Özdemir and Kavak Budak (2022)	I = MBSR	The program included MBSR meditations (e.g., mindful breathing, eating, daily activities), basic information on schizophrenia and stress, and habit-breaking exercises (e.g., nature time, sky watching, thought-emotion games, writing a letter to self, and random acts of kindness).
	C = TAU	CMHCs provide daytime rehabilitation for individuals with severe mental illnesses post-hospitalization, offering psychosocial skills training and support. Facilities include reading, sports, training, and dining rooms, staffed by psychiatrists, psychologists, nurses, and assistants.

Notes. CBT = Cognitive Behavioral Therapy, CMHC = Community Mental Health Center, ECAU = enhanced care as usual, GAP = Goal Setting and Planning Skills, I-CAT = Integrated Coping Awareness Therapy, IMR = Illness Management and Recovery, MBSR = Mindfulness-Based Stress Reduction, PPI = Positive Psychology Intervention, TAU = Treatment As Usual.
